# The Computer Simulation for Triggering Anxiety in Panic Disorder Patients Modulates the EEG Alpha Power during an Oddball Task

**DOI:** 10.3390/neurosci3020024

**Published:** 2022-05-31

**Authors:** Luiza Di Giorgio Silva, Danielle Aprigio, Victor Marinho, Silmar Teixeira, Jesse Di Giacomo, Mariana Gongora, Henning Budde, Antonio E. Nardi, Juliana Bittencourt, Mauricio Cagy, Luis Fernando Basile, Marco Orsini, Pedro Ribeiro, Bruna Velasques

**Affiliations:** 1 Neurophysiology and Neuropsychology of Attention Laboratory, Institute of Psychiatry, Federal University of Rio de Janeiro, Rio de Janeiro 22290-140, Brazil; luizadigiorgio@gmail.com (L.D.G.S.); danyaprigio@gmail.com (D.A.); marianagongora@gmail.com (M.G.); juju_bitt@yahoo.com.br (J.B.); bruna_velasques@yahoo.com.br (B.V.); 2 Neuro-Innovation Technology & Brain Mapping Laboratory, Federal University of Delta do Parnaíba, Parnaíba 64202-020, Brazil; silmarteixeira@ufpi.edu.br; 3 Brain Mapping and Sensory Motor Integration Laboratory, Institute of Psychiatry, Federal University of Rio de Janeiro, Rio de Janeiro 22290-140, Brazil; silva.jesse@gmail.com (J.D.G.); ribeiropss@yahoo.com.br (P.R.); 4 Institute of Applied Neuroscience, Rio de Janeiro 22290-140, Brazil; 5 Faculty of Human Sciences, Medical School Hamburg, 20457 Hamburg, Germany; henning.budde@medicalschool-hamburg.de; 6 Laboratory of Panic & Respiration, Federal University of Rio de Janeiro, Rio de Janeiro 22290-140, Brazil; antonioenardi@gmail.com; 7 Department of Physiotherapy Rio de Janeiro, Veiga de Almeida University, Rio de Janeiro 20271-901, Brazil; 8 Biomedical Engineering Program, Federal University of Rio de Janeiro, Rio de Janeiro 20271-901, Brazil; mauricio.cagy@gmail.com; 9 Division of Neurosurgery, University of São Paulo Medical School, São Paulo 01246-904, Brazil; lbasile@gmail.com; 10 Master’s Program, Vassouras University, Vassouras 27700-000, Brazil; orsinimarco@hotmail.com

**Keywords:** panic disorder, EEG alpha power, oddball paradigm, computer simulation, working memory

## Abstract

Aim: The present study investigated the differences between the Panic Disorder (PD) patients groups’ and healthy controls for the EEG alpha dynamics under the frontal cortex and reaction time during the oddball task. Material and Methods: The reaction time during the oddball paradigm concomitant to EEG alpha power was tested in nine PD patients and ten healthy controls before and after a computer simulation presentation. Results: The findings revealed a decrease in EEG alpha power in PD patients concerning the control group (*p* ≤ 0.0125). However, both groups demonstrated an increased cortical oscillation after the computer simulation, except for the Fp1 electrode during M3 moment in the experimental group. The experimental group has a fast reaction time compared to healthy individuals during the oddball task (*p* = 0.002). Conclusions: We propose that the decrease in EEG alpha power in the PD patients may indicate an increase in processing related to an anxiogenic stimulus and interference of the anxiety state that compromises the inhibitory control. The reaction time task reveals cognitive symptoms in the experimental group, which may be related to the faster reactivity and high impulsivity to stimuli.

## 1. Introduction

The anxiety disorder that culminates in panic disorder (PD) is a multidimensional disorder that involves the activation of complex brain circuitry, characterized by repeated and unexpected attacks of intense anxiety, not restricted to a determined circumstance, and which can result in tachycardia, breathlessness, asphyxia, fear of death, fear of losing control, and others [1,2,3]. Different models of panic disorder have been proposed, such as the cognitive [2,3,4], learning [5], and biological models [6], but the neurobiological theory of PD is not completely clear yet [7]. According to Howe et al. [8], there is converging evidence about the role of defective data/sensory processing and maladaptive attention span involved in PD’s neurophysiology.

Previous studies through electroencephalography (EEG) and brain imaging in the PD patients evidenced that it has coherence between the cognitive processing and neurobiological hypotheses, which attributes the PD anxiety to a dysfunctional interaction between the prefrontal cortex and the limbic system [9,10,11,12]. According to Gorman et al. [6], a disability in the coordination of stimuli from the frontal cortex and brainstem could engender an abnormal activation of the amygdala, with a behavioral, autonomic, and neuroendocrine stimulation. Amygdala activation may be a consequence of misinterpretation of sensory information, and it allows the cascade of neural events related to a panic attack [9].

These neuroanatomical activation differences observed in PD patients led to the following question: What are the cognitive deficits related to PD? The systematic review conducted by O’Sullivan and Newman [13] indicated an absence of difficulties in PD patients relative to the control group. However, the authors pointed out that there was some support for potential impairments during reaction time tasks in short term verbal and visual memory execution compared to healthy individuals. Simultaneously, the findings of the working memory performance in PD patients are controversial. Some of them showed no impairments [14,15], while others point to an impairment in this cognitive function [16]. This can be related to difficulties in information processing and, consequently, to the misinterpretation of bodily symptoms. Dratcu and Bond [16] explained that deficits in working memory and explicit memory might be related to a high level of excitement and anxiety in patients during the task execution.

Nowadays, neuroscience researchers have been using computer simulations to observe neurobiological parameters during and after virtual contact with phobic environments or situations. In this context, the anxiety related to stimuli observed on the video can trigger anxiety symptoms, such as body sensations, alteration of cognitive task performance, and neural inputs during task processing. This technique allows the investigation of induced anxiety with PD patients in an environment fully controllable by the investigator [17]. Virtual reality is a useful psychotherapy tool focused on anxiety disorders [18], such as agoraphobia, and specific phobias, such as driving phobia, as a mechanism for exposure therapy [19].

Additionally, brain imaging studies in PD patients have found neural deficits during neurotransmission in some cortical and subcortical areas, namely the frontopolar cortex (FPC), dorsolateral prefrontal cortex (DLPFC), ventrolateral prefrontal cortex (VLPFC), thalamic nuclei, and amygdala [20,21,22]. Among these areas, evidence demonstrates that DLPFC and VLPFC are essential for cognition and executive functions such as inhibitory control, motor inhibition, visual processing, and perception, all built for decision making for cognitive tasks [23]. For this reason, cortical and subcortical changes are implicated in many PD symptoms [24]. Neurocognitive factors elucidated by behavioral tests and EEG-analysis indicate that PD patients may have functional visual stimuli deficit [10]. Previous studies showed that these neural networks participate in impulsive behavior, which is defined as a premature response and instantaneous gratification, performed before all available information. With this in mind, impaired timing functions are the key to PD’s behavioral profile [25].

Neurophysiological studies demonstrated that ongoing brain oscillations are linked to the perceptual system’s intrinsic tendency to process information within different temporal windows. The activity in the EEG alpha band (8–12 Hz) range is suggested to be a correlation in neurobiological aspects inbuilt in the decision making for cognitive strategies and neurobiological aspects (memory, attention, and visual perception) [26,27]. As the EEG has been used for the functional network analysis in the topological changes associated with cognitive regions in real-time [18,28,29,30], we aimed to observe the computer simulation effects on EEG alpha power and the reaction time for visual stimuli in the PD patients. Our electrophysiological variable of interest is the alpha absolute power due relationship with the top down and inhibitory processes [31,32,33,34]. This trait deficit in alpha activity has been proposed as a risk factor for several psychiatric disorders, including anxiety disorders [35].

EEG alpha power has been used in psychiatric research, investigating cognitive processing impairments over anxiety disorders [19,32,33,36]. Thus, few studies have analyzed the correlation among PD, EEG alpha power, and the oddball paradigm. This context could be relevant to describe the interference of anxiety on the brain areas’ communication and how it can affect information processing. Furthermore, a large portion of computer simulation potential regarding PD research is still unexplored [19].

In this context, we hypothesized that PD patients would present differences in the frontal activation during the oddball task compared with healthy controls expressed by EEG alpha power, mostly after high anxiety (produced by computer simulation). The increase in the EEG alpha power in anxiety support our hypothesis [9,32,33]. Therefore, the aims of the present study are (1) to analyze the differences in the EEG alpha power during the oddball task between experimental and control groups before and after computer simulation; (2) to verify if the high levels of anxiety affect the reaction time of subjects with PD before and after watching an anxiogenic computer simulation [37].

## 2. Materials and Methods

### 2.1. Participants

Sample size was calculated based on a cross-sectional study that applied the same experiment to assess the EEG dynamics under cortical areas and reaction time during the oddball task [10]. The GPower 3.1 software (Heinrich-Heine-Universität Düsseldorf) was used to calculate the sample size, with an alpha level of 0.05 and a power of 90%, based on standard deviations and differences between the means obtained in the study by Silva et al. [10].

Nineteen subjects, residents in the state of Rio de Janeiro with age from 25 to 60 years (42.35 ± 12.04 years), performed the oddball task and computer simulation analysis. The sample was composed of a control group (10 healthy subjects, 1 man and 9 women, 38.2 ± 13.69 years), and an experimental group from the Psychiatry Institute of the Federal University of Rio de Janeiro (9 women with panic disorder, 48.8 ± 11.16 years).

The patients were examined by doctors to confirm that they had the 13 symptoms used in the diagnosis criteria of a panic attack in DSM-IV [1]. Second, psychosomatic medicine specialists excluded physical diseases such as arrhythmia, angina, hyperthyroidism, chronic obstructive pulmonary disease (COPD), asthma, pheochromocytoma, and neurological disorders, including evident epilepsy. Additionally, the participants were under psychiatric treatment. To avoid the pharmacological bias, they were asked to suspend medication one day before the exam. The patients who regularly take psychotropic drugs and other medicines with EEG effects were excluded, such as patients with schizophrenia, severe depression, and personality disorders; patients with alcoholism or drug abuse; and patients with severe circulatory, respiratory complications, digestive, endocrine, and neurological disease [38].

Both control and experimental groups were selected based on the Edinburgh Lateral Dominance Inventory, adapted from Oldfield (1971) [39]. All participants underwent a medical evaluation to exclude those with other neurological or motor diseases or visual, hearing, and motor impairments that would impair the task’s performance.

The healthy participants were instructed not to use any substance that can inhibit or stimulate brain activity (e.g., tobacco, coffee, alcoholic beverages, foods containing caffeine, and medications) 24 h before task time.

The groups were matched by age. An Independent T-Test was performed between the two groups and showed no significant difference between these two groups for age range (*p* > 0.05). All subjects provided written informed consent before entering the study, according to the Declaration of Helsinki.

### 2.2. Experimental Procedure

The experiment happened in a room with controlled lighting and temperature and with electrical and sound insulation. The control and experimental groups sat down on a chair with armrests to minimize any muscular artifact during EEG signal acquisition. EEG data were collected before, during, and after the oddball task. All procedures were the same for both groups.

**Figure 1:**
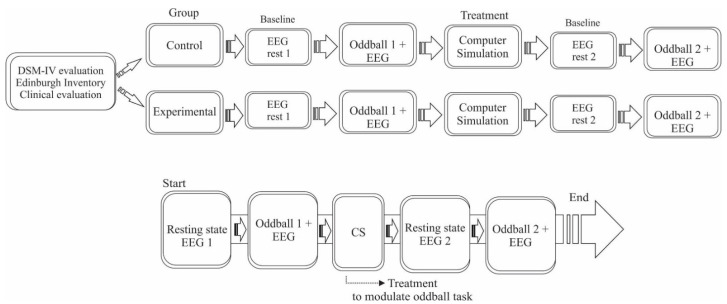
Illustration of the experimental procedure.

Individuals sat in front of a 28′ monitor. First, a resting state EEG (3 min) was performed to determine the baseline. After the resting state EEG, the oddball task (oddball task 1) was performed concomitant to EEG recording. Following the procedure, we inserted computer simulation as an anxiety inducer for four minutes (the treatment was used to determine if they modulated the oddball task performance). Subsequently, after the computer simulation, a resting state EEG (3 min) was performed, and finally, the oddball task (oddball task 2) was associated with EEG recording (Figure 1). The objective was to observe how anxiety affects the information processing and working memory of PD patients. The visual stimulus was presented on the monitor by the Event-Related Potential (ERP) Data Acquisition Software (Brain Mapping and Sensorimotor Integration Laboratory, Rio de Janeiro, Brazil), developed in Delphi 5.0 (Borland Developer Inprise Co. Microsoft, Austin, TX, USA). The experiment was conducted in the Electrophysiology and Neuropsychology of Attention Laboratory.

### 2.3. Visual Oddball Task

**Figure 2:**
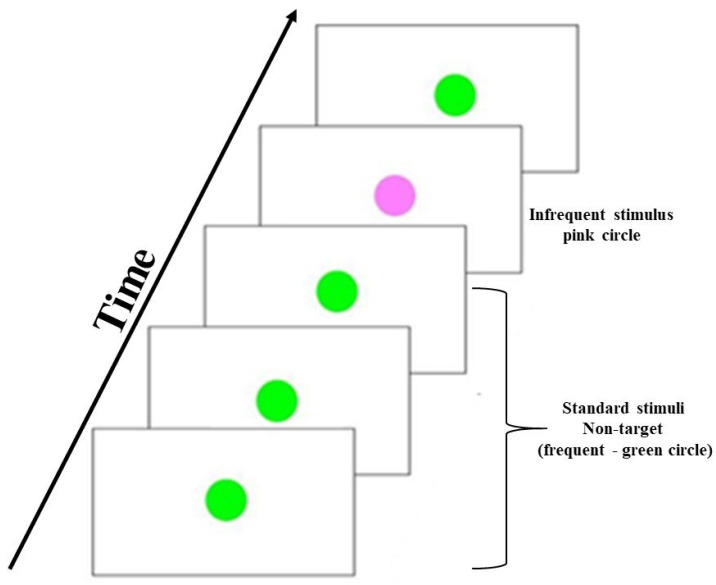
The temporal sequence of the oddball visual task. The green-colored circle stimulus is the “frequent” or “standard” stimulus. The pink-colored circle is the “rare” or “deviant” or “target” stimulus. The number of standards presented between two deviants is pseudo-random [40].

A fundamental paradigm for evaluating the time reaction with visual stimulus, referred to as the “Oddball” paradigm, contains a rare (or “deviant”) target stimulus presented along with more frequent (or “standard”) non-target stimuli in a serial input stream (Figure 2). The oddball task is a useful method to evaluate information processing, event-related potential, and reaction time [7,40]. The Visual Oddball paradigm consists of two stimuli presented randomly, with one of them occurring relatively infrequently. The stimuli randomization of the oddball visual task has as main objective to avoid any practical effect related to the task’s learning. The subjects need to discriminate target (infrequent, pink circle) from non-target or standard stimuli (frequent, green circle). Subjects were instructed to respond as quickly as possible to the target stimulus by pressing a button on a joystick (Model Quick Shot- Crystal CS4281). Each stimulus lasted 2.5 s, being the same interval time between stimuli. Each subject performed 2 blocks with 40 trials each.

In the oddball task, the repetition standard numbers between two occurrences of a (target) deviant are randomized, such that the length of the sequence of interest is random. The perceiver is thought to “compute online” a conditional probability of the target occurrence. However, as the number of consecutive standards increases, the probability of occurrence of the target increases too, which increases the likelihood of a motor response requirement, hence affecting: (1) the level of attention and/or motor decision mechanisms, and (2) the amount of motor preparation [40].

### 2.4. Computer Simulation

**Figure 3:**
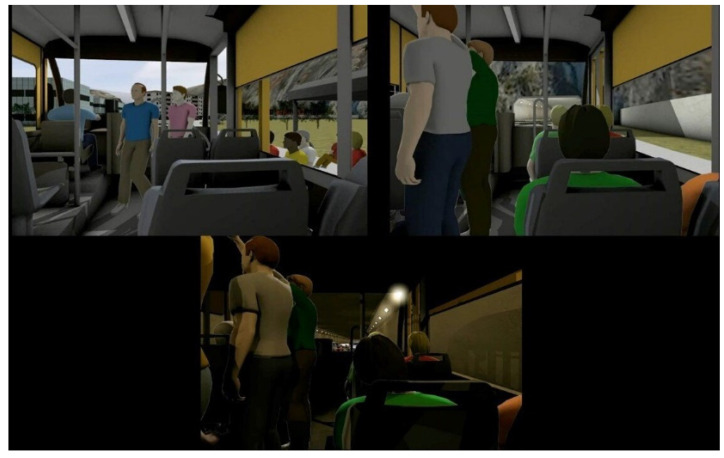
Illustration of the computer simulation. The subjects were instructed to look at the center of the screen at all times. The animation was in a first-person perspective, and camera movement occurred as if the subject was walking and looking in different directions. The computer simulation for triggering anxiety is cognition-mediated technique that bridges still images and virtual reality. In the context, images are easy to manage and can be anxiogenic/panicogenic. On the other hand, it can be tailored to the patient’s needs, is interactive, induces meaningful sense of presence, and can to compare the effects of virtual reality sessions.

Previous studies confirmed that the simulation is a useful method to induce anxiety [9,37]. It was a 4 min, three-dimensional computer animation developed by TriptyqueLAB (www.triptyquelab.com accessed on 19 March 2022) (Figure 3). The computer simulation for 4 min consisted of 30 s of white screen, followed by 3 min of anxiogenic situations, and then more 30 s of a white screen. The animation was in a first-person perspective and started at a bus stop, and the bus arrives. The subject boards and sits on the bus, the bus moves through city streets, stops again. The bus fills with people, moves through the streets, enters a tunnel, stops inside the tunnel due to traffic, starts moving again, leaves the tunnel, and stops at a bus stop. The subject leaves the bus and watches the bus drive away [37,41]. The simulation included sounds related to the context of the images. The subjects were exposed to computer simulation just once.

### 2.5. EEG Recording

The room was free of acoustic insulation, electrical grounding, and low light. Subjects sat in a chair with armrests to minimize muscle artifact during EEG signal acquisition. The 20-channel continuous EEG was recorded by BrainNet BNT36 (EMSA Medical Equipment – Rio de Janeiro -Brazil). The silver/silver chloride electrodes were positioned through a nylon cap following the international 10–20 system, including binaural reference electrodes (SPES Medical Brazil). The EEG electrodes impedance and electrooculogram (EOG) electrodes were kept below 5 kΩ. The acquired data had an amplitude below 100 μV. The sampling rate was 240 Hz. An anti-aliasing low-pass filter with a cut-off frequency of 100 Hz was employed. Its configuration uses 60 Hz Notch digital filtering, with high-pass filters at 0.1 Hz and low pass filters at 40 Hz (Order 2 Butterworth filter), using the Data Acquisition software (Delphi 5.0 - Microsoft USA ) developed in the Brain Mapping and Sensorimotor Integration Laboratory. The signal corresponding to each EEG derivation came from the electric potential difference between each electrode and the pre-set reference (earlobes). The epochs were time-locked to the stimulus presentation, and we extracted 4 s before and 4 s after the stimulus. Each subject had 20 epochs.

#### Electrodes of Interest

We selected the Fp1, Fp2, F3, F4, F7, and F8 electrodes for measure the EEG alpha power (8–12 Hz) due to the relationship with the frontopolar cortex, dorsolateral prefrontal cortex, and ventrolateral prefrontal cortex, respectively. Both are related to working memory, attention, sensorimotor orientation, preparation, and motor response inhibition [42,43]. We selected the electrodes based on the capability of sensorimotor integration in visual–spatial processing, coordination, and modulating the attentional level for multimodal perception [44].

### 2.6. Data Processing

A visual inspection and independent component analysis (ICA) was applied to identify and remove all remaining artifacts through MATLAB version 12.0.2b (The Mathworks, Inc., Natick, MA, USA). Data from individual electrodes that showed contact loss with scalp or high impedance (>5 kΩ) were not considered. The overall rate of removal after ICA was less than 10%. Only the remaining epochs were part of subsequent signal processing and statistical analysis. A classical estimator (i.e., parametric, Bartlett Periodogram, using non-overlapping 2 s long (480 samples) rectangular windows) was applied to the Power Spectral Density (PSD), estimated from the Fourier Transform (FT), which was performed using MATLAB [45,46]. Afterward, stimuli locked epochs were computed, comprising an interval of −0.5 s to +1.5 s corresponding to stimulus presentation. Trials were then baseline-corrected. Data from single-trial epochs exhibiting excessive movement artifact (±100 μV) were also deleted [9,10].

### 2.7. Statistical Analysis

We performed a Two-Way Mixed ANOVA to analyze the differences of the EEG alpha power for each electrode separately: Fp1, Fp2, F3, F4, F7, and F8, with factors: group (control vs. experimental) and moments (M1- resting-state EEG vs. M2- EEG-oddball 1 vs. M3- resting-state EEG after CS vs. M4- EEG-oddball 2 after CS). Additionally, we performed a Two-Way Mixed ANOVA to analyze the differences for reaction time, with factors group (control vs. experimental) and moment (oddball 1 vs. oddball 2). All results are given as mean and standard error (SE). The interaction analysis was performed using the Independent *t*-test, and a one-way ANOVA was used between the group and within the moment’s analysis. Multiple corrections were made by the Scheffe test for possible behavioral and neurophysiological interactions, considering the *p* ≤ 0.0125 (0.05/4).

We used the Mauchly’s test criteria to evaluate the sphericity hypothesis and the Greenhouse–Geisser (G-Gε) procedure to correct freedom degrees. The normality and homoscedasticity of the data were previously verified by the Shapiro–Wilk and Levene tests. The effect size was estimated as partial eta-squared (ƞ^2^p) in mixed factorial ANOVA. Additionally, the analysis effect was evaluated by Cohen’s *d* for the Student *t*-test. Statistical power and the 95% confidence interval (95% CI) were calculated for the dependent variables. Statistical power was interpreted as the low power of 0.1 to 0.3; high power from 0.8 to 0.9. The effect magnitude was interpreted using the recommendations suggested by Cohen (1998): insignificant <0.19; small from 0.20 to 0.49; mean from 0.50 to 0.79; large from 0.80 to 1.29 [47]. We adopted the probability of 5% for type I error for all analyses (*p* < 0.05). The analyses were conducted in SPSS for Windows version 20.0 (SPSS Inc., Chicago, IL, USA).

## 3. Results

### 3.1. EEG Alpha Power Analysis

**Figure 4:**
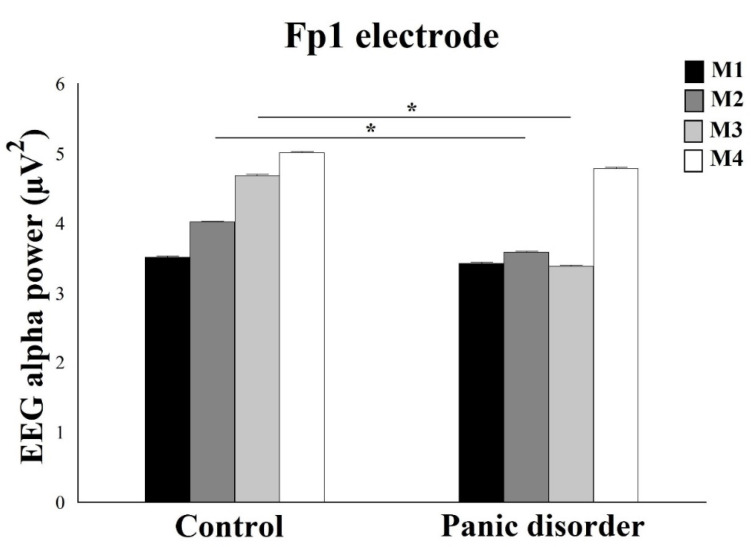
In relation to the Fp1 electrode, the analysis shows a decrease in the EEG alpha power in the experimental group when compared to control for moment M2 and M3. The interaction between group and moment for the left frontopolar cortex is demonstrated by mean ± standard error, and statistically significant difference is indicated with *, *p* ≤ 0.0125.

A Two-Way Mixed ANOVA for Fp1 electrode showed interaction for Group and Moments (F(4) = 4.75; *p* = 0.003; ƞ^2^p = 0.51; power = 98%) (Figure 4). When analyzing the interaction by One-way ANOVA within each Group, control showed a statistical difference for Moments (F(2) = 7.87; *p* = 0.001; ƞ^2^p = 0.53; power = 96%), with Scheffé post hoc test demonstrated a difference between M4 and M1, and M4 and M2 (*p* = 0.002). The findings showed that the M4 produced a high EEG alpha power concerning other moments. In the experiment, the results evidenced a statistical difference for Moments (F(2) = 7.44; *p* = 0.005; ƞ^2^p = 0.52; power = 98%), with difference among M4 and the other M1, M2, and M3 moments (*p* ≤ 0.0125). The findings showed that the M4 produced a high EEG alpha power concerning other moments. The analysis between groups by independent *t*-test for EEG alpha power under F1 electrode showed statistical difference for M2 (*t*(2) = 6.57; *p* = 0.0001; *d* = 0.55) and M3 (*t*(2) = 8.45; *p* = 0.0001; *d* = 0.63), with a decrease in EEG alpha power in the experimental group when compared to control (*p* = 0.002).

**Figure 5:**
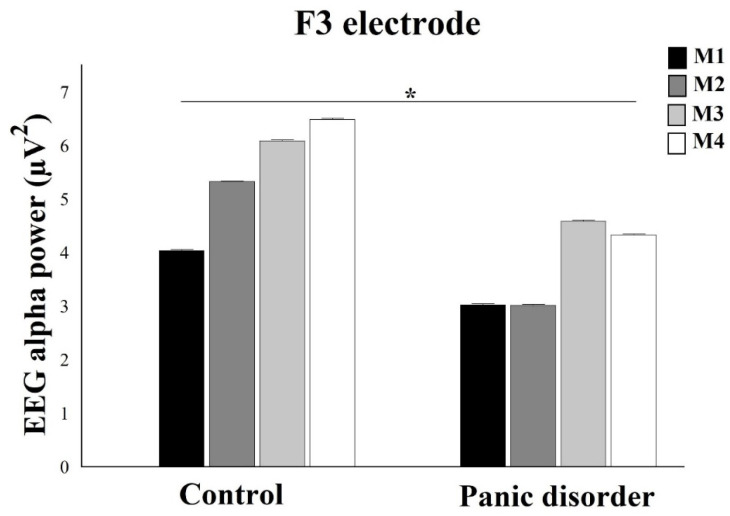
In relation to F3 electrode, the analysis shows differences between groups for all moments. The EEG alpha power decreases in the experimental group when compared to control. The interaction between group and moment for the left dorsolateral prefrontal cortex is demonstrated by mean ± standard error, and statistically significant differences are indicated with *, *p* ≤ 0.0125.

A two-way mixed ANOVA showed interaction between Group and Moments for F3 electrode (F(4) = 6.76; *p* = 0.004; ƞ_2_p = 0.56; power = 96%) (Figure 5). When analyzing the interaction by One-way ANOVA within each group, results showed difference for control, with (F(2) = 24.13; *p* = 0.0001; ƞ^2^p = 0.52; power = 100%). The Scheffé post hoc showed a difference between M4 and M1 and between M4 and M2 (*p* < 0.0001). We observed that M4 produced an increase in EEG alpha power compared to M1 and M2. In addition, for experimental was evidenced statistical difference, with (F(2) = 8.45; *p* = 0.002; ƞ^2^p = 0.53; power = 97%). The Scheffé post hoc reveals a difference between M3 and M1 and between M3 and M2 (*p* = 0.0001). The M3 had greater EEG alpha power when compared to M1 and M3. The analysis between groups by independent t-test, for EEG alpha power under F3 electrode, evidenced differences for all the moments (*p* ≤ 0.0125), with an increase EEG alpha power after computer simulation.

**Figure 6:**
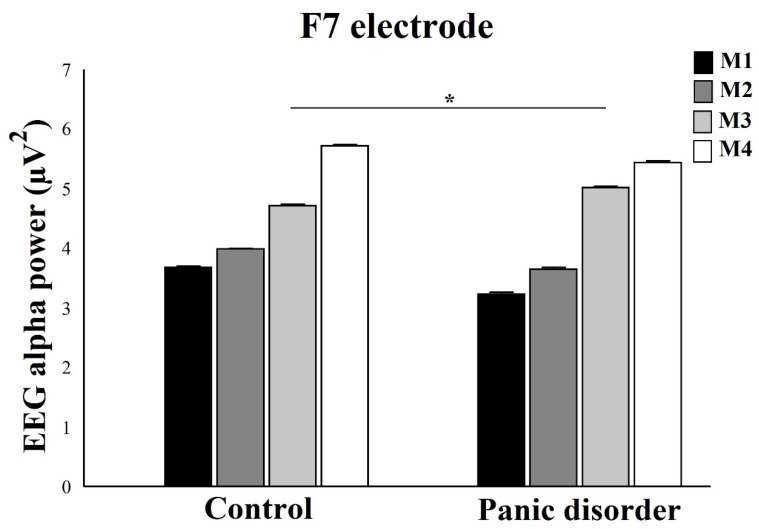
In relation to F7 electrode, the analysis shows an increase in the EEG alpha power in the experimental group when compared to control for M3 moment. The interaction between group and moment for the left ventrolateral prefrontal cortex is demonstrated by mean ± standard error, and statistically significant difference is indicated with *, *p* ≤ 0.0125.

A two-way mixed ANOVA evidenced interaction between Group and Moments for F7 electrode (F(4) = 7.11; *p* = 0.0006; ƞ_2_p = 0.58; power = 99%) (Figure 6). When analyzing the interaction by One-way ANOVA within each group, results showed a difference for control, with (F(2) = 8.73; *p* = 0.0001; ƞ^2^p = 0.50; power = 100%). The Scheffé post hoc showed a difference between M4 and M1 and between M4 and M2 (*p* = 0.001). We observed that the M4 produced an increase in EEG alpha power compared to M1 and M2. In addition, the experiment showed a statistical difference, with (F(2) = 10.05; *p* = 0.0002; ƞ^2^p = 0.54; power = 99%). The Scheffé post hoc reveals a difference between M4 and M1 and between M4 and M2 (*p* = 0.001). M4 had greater EEG alpha power when compared to M1 and M3. The analysis between groups by independent t-test, for EEG alpha power under F3 electrode, only showed differences for M3, with (*t*(2) = 9.05; *p* = 0.001; *d* = 0.51), with an increase EEG alpha power after computer simulation.

**Figure 7:**
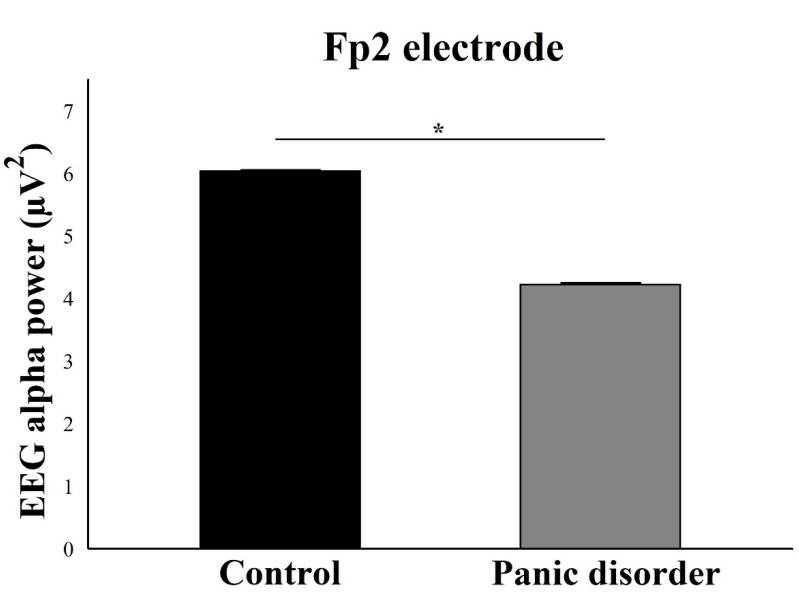
Main effect for group (*p* = 0.002). The EEG alpha power for Fp2 electrode decreases in the experimental group compared to the control group. The result is represented by the mean ± standard error and the statistically significant difference is indicated with *, *p* < 0.05.

**Figure 8:**
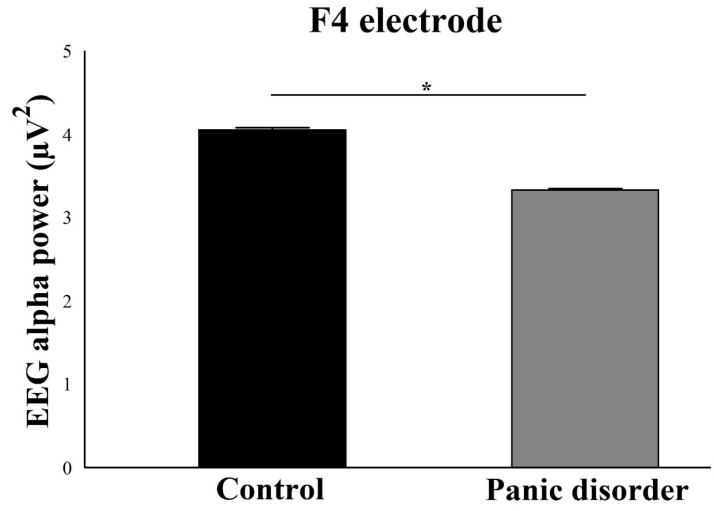
Main effect for group (*p* = 0.001). The EEG alpha power for F4 electrode decreases in the experimental group compared to the control group. The result is represented by the mean ± standard error and the statistically significant difference is indicated with *, *p* < 0.05.

**Figure 9:**
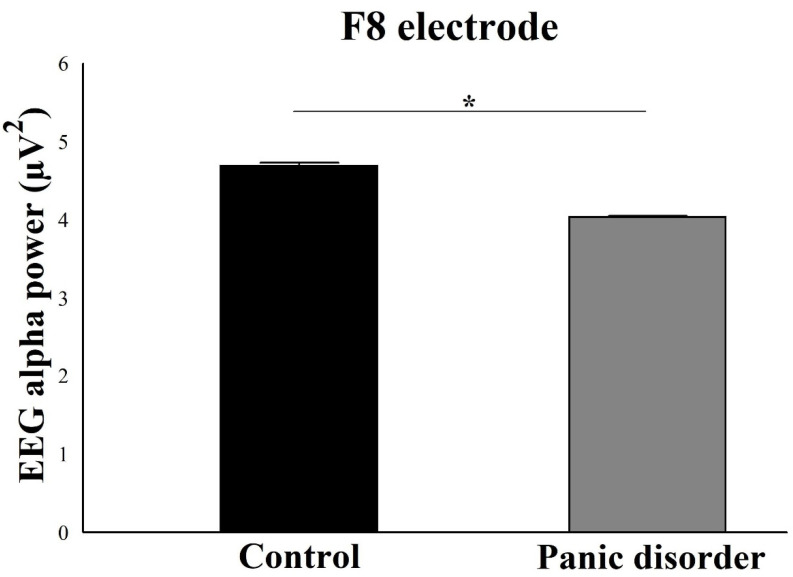
Main effect for group (*p* = 0.001). The EEG alpha power for F8 electrode decreases in the experimental group compared to the control group. The result is represented by the mean ± standard error and the statistically significant difference is indicated with *, *p* < 0.05).

For the electrodes of the right hemisphere, the results suggested a main effect for Group for the electrodes Fp2 (F(2) = 6.03; *p* = 0.002; ƞ^2^p = 0.48; power = 97%) (Figure 7), F4 (F(2) = 7.35; *p* = 0.001; ƞ^2^p = 0.51; power = 99%) (Figure 8), and F8 (F(2) = 4.41; *p* = 0.001; ƞ^2^p = 0.46; power = 95%) (Figure 9). In general, there was a decrease in the EEG alpha power in the experimental group.

### 3.2. Reaction Time

**Figure 10:**
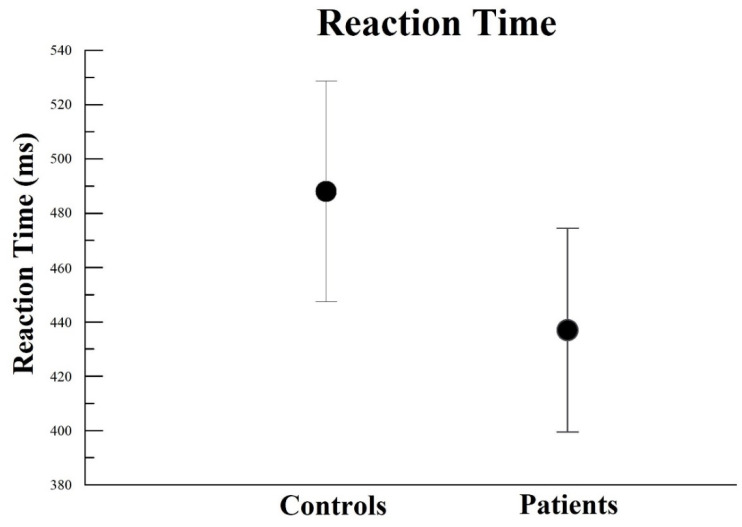
Main effect for group (*p* = 0.002). Modification of the reaction time in relation to computer simulation for trigger anxiety. The result is represented by the mean ± standard error.

The findings of reaction time demonstrated no interaction by two-way mixed ANOVA (*p* > 0.05). However, there was a main effect for Group, with (F(2) = 10.73; *p* = 0.002; ƞ^2^p = 0.58; power = 98%) (Figure 10). We observed that HC control (average: 488.06 ± 40.67 ms) was slower than PD patients (average: 437.01 ± 37.55 ms). It is important to say that the experimental group’s RT performance was high compared to the control.

## 4. Discussion

This present study investigated the EEG alpha power over the frontal cortex and reaction time in PD patients versus healthy controls during the oddball task before presenting a computer simulation that triggers anxiety symptoms. Our main result indicated a decreased alpha power in PD patients, however demonstrated an increase in EEG alpha power after the computer simulation in both groups, except for the Fp1 electrode during M3 moment in the experimental group. According to previous studies, this result reported a low EEG alpha power in PD patients [9,32,33,34,48,49]. Previous studies demonstrated a greater EEG alpha power for healthy subjects was found when compared to the PD patients in the frontal area. The greater frontal activation could be related to an impaired frontal attempt to regulate downstream excitability or reflect the excitation from deeper subcortical regions [9].

If the frontal cortex top-down modulation is not working correctly, anxiety symptoms are likely to be more prominent [9,50]. The low EEG alpha power for the left frontopolar cortex, left dorsolateral prefrontal cortex, and left ventrolateral prefrontal cortex we found is also in line with the possibility of an impaired top-down regulation and inhibition control [51,52]. Our findings indicate that the decreased EEG alpha power may reflect a dysfunction in thalamic–cortical circuits associated with incapacity to inhibit irrelevant information [31,49]. Thus, the PD patients may present impairment both in inhibitory control and top-down regulation during the anxiety state, related to the state of high excitability and lower inhibitory control [33,50].

While the decrease in EEG absolute power represents a high excitability state [33], the higher EEG alpha power has been evidenced after the computer simulation in both groups, except for the Fp1 electrode during M3 moment in the experimental group. This way, our result of greater EEG alpha power after computer stimulation may trigger a dysfunctional activation and regulation of excitability over the frontal, parietal, and temporal PD patients’ networks. In a systematic review, Di Giorgio et al. [53] evidenced that many PD researchers noted that PD patients present impairment in information processing after stimuli that cause emotional discomfort, which could be related to a failure to automatically inhibit responses to fear or failure in the modulation of more sophisticated and conscious responses [53].

The results presented by PD patients of higher EEG alpha power in the M3 and M4 (after the film presentation) in the F3 electrodes point to the anxiogenic film’s influence, increasing the anxiety and the information processing during the task. This way, these patients could have impairment in working memory’s accuracy to execute the oddball task after the CS exposition. It is excessive cognitive processing, but this phenomenon resembles a kind of emotional blindness when acute anxiety interferes with information processing and descriptive ability [33,50,54,55].

Another significant result of this research was the difference between the reaction time. The main effect minimizes inferences regarding computer simulation; however, the behavioral results corroborate impulsivity symptoms for the visual stimulus during the oddball task. We believe that it may be deficit synchronization of stimuli when the participant should use cognitive functions of information processing to remember the target stimuli, make comparisons between the target and non-target stimuli, and react by pressing the button if the target stimuli were presented or inhibit this behavior if the non-target were presented [9].

The inhibitory control, impaired in PD patients, alters the executive functions (e.g., executive control of behavior, inductive reasoning, and planning), visual and motor working memory, and visuospatial processing during decision making for task [15,16]. In support of the neuro-physiological finding, the emotional blindness during decision-making can affect the task execution, just as these patients can experience in their ordinary lives difficulty to perceive, process, and discriminate information when under the influence of anxiety.

PD patients were faster than healthy individuals during the oddball task. This leads us to question if the faster reactivity to PD patients could be related to the high excitability to new stimuli and classic symptoms of the disorder. In a perception limits paradigm, PD patients present a significantly faster reaction time when they identify panic-related words next to neutral words. This result could indicate an explicit memory bias of PD patients for anxiety [3], which may be related to the cognitive model of time perception in neurological diseases, explaining the dysfunctional misinterpretation of external stimuli [3] and bodily sensations [2] as signs of confirmation of the present danger, potential situation, or stimuli.

What is the role of the left frontal region in this working memory context after contact with a CS movie that induces anxiety? The information needs to be processed and sent to the frontal region for decision making. This interference of higher alpha power after computer simulation could be related to PD patients’ abnormal processing [56,57,58,59]. Goldstein [56] showed that damage to the left hemisphere was more likely to cause a catastrophic-depressive reaction in psychiatric patients than damage to the right hemisphere. The left brain hemisphere is associated with emotional processing, and we assumed that negative emotions affected the alpha band activity in this region due to the execution of a cognitive task influenced by an anxiogenic movie, affecting behavioral performance.

This study has some limitations, which include sample size. However, the statistical power in the analysis decreases the possibility of a type I error. Other limitations include the non-evaluation of Electromyography (EMG), as the participants were instructed to avoid random moving during the oddball task; however, this was not confirmed with EMG to measure muscle activity. We could also have used instruments to assess attentional level and perception to relate both reaction time interpretation with the activity of the prefrontal cortex.

## 5. Conclusions

Our findings confirm that PD patients present a lower EEG alpha power than healthy controls and the influence of anxiety triggering stimulus on cortical oscillations by the anxiogenic situation. The increase in the EEG alpha power in the M3 and M4 (corresponding to the moment after computer simulation) can reflect this movie’s anxiogenic potential as it increases the anxiety and information processing. The reaction time results showed that PD patients were faster than the control group in the oddball task, which could be related to the high cortical excitability and impulsivity symptomatology. The findings pinpoint the interference of anxiety in reaction time and loss of precision. The excitement and anxiety could lead to a loss of selective attention, which mediates encoding information and support received. Thus, these patients have a faster reactivity that is not accompanied by the accuracy of the information processing and could be explained by the fact that impulsivity is higher in patients with anxiety disorders.

## Data Availability

Not applicable.
